# Fecal Sample Surveillance of the Wildlife Virome in Central Italy: Insights from the Foreste Casentinesi National Park

**DOI:** 10.3390/ani15233378

**Published:** 2025-11-21

**Authors:** Maria Irene Pacini, Mario Forzan, Maurizio Mazzei

**Affiliations:** Department of Veterinary Sciences, University of Pisa, 56124 Pisa, Italy; mariairene.pacini@phd.unipi.it (M.I.P.); maurizio.mazzei@unipi.it (M.M.)

**Keywords:** wildlife virome, non-invasive surveillance, next-generation sequencing

## Abstract

Wildlife can act as both a reservoir and a sentinel for emerging pathogens, but surveillance is often constrained by difficulties in obtaining samples without disturbing animals. This study explored the viral diversity of wild mammals inhabiting the Foreste Casentinesi National Park (Central Italy) using non-invasive fecal sampling. From 2021 to 2022, 99 fecal samples from several species were collected and analyzed by PCR and metagenomic next-generation sequencing. Of 26 pools examined, 10 (38.5%) tested positive for at least one viral target. Astroviruses were the most frequently detected, found in deer, foxes, wolves, small mustelids, and porcupines. Several sequences showed low similarity to known strains, suggesting divergent or novel viral lineages. Metagenomic analysis also identified members of *Circoviridae*, *Anelloviridae*, and *Picobirnaviridae*. While none of these virus families are currently recognized as major zoonotic agents, their widespread occurrence in wildlife and domestic animals underscores the importance of continued surveillance to better assess their ecological roles, host range, and potential implications for both animal and human health. These results provide new insights into the virome of European wildlife, including the first reports of some viruses in certain species. Overall, our study demonstrates that non-invasive surveillance is a valuable tool for monitoring ecosystem health and supports a One Health approach to early detection of viral threats.

## Simple Summary

Wild animals can host many viruses, some of which may also affect humans and domestic animals. Monitoring these viruses is challenging because capturing wild animals is stressful and often impractical. In this study, we collected feces left in the environment by several mammal species living in the Foreste Casentinesi National Park in Tuscany, Italy. Using laboratory methods able to detect both known and unknown viruses, we found that more than one-third of the samples contained viral genetic material. Astroviruses were the most common and were detected in deer, foxes, wolves, porcupines, and small mustelids. Foxes carried the highest variety of viruses, such as parvovirus, coronavirus, and adenovirus. Because predators and scavengers may also ingest viruses from their prey, fecal samples provide information not only on individual animals but also on the broader community. Our results show that non-invasive sampling can give important insights into wildlife health, help identify new viruses early, and support biodiversity protection and public health.

## 1. Introduction

Wildlife represents an essential component of European biodiversity, contributing to the ecological, structural, and functional balance of natural ecosystems. However, recent assessments indicate that approximately 63% of protected animal species in Europe are unable to maintain viable populations, while 81% of natural habitats remain in unfavourable conservation status due to habitat loss, fragmentation, and degradation and the threat of infectious diseases [1]. When natural habitats are destroyed and biodiversity declines, humans and wildlife come into closer contact, increasing the risk of pathogen spillover. Conversely, conserving healthy ecosystems and diverse species can help regulate disease transmission and prevent outbreaks. In this way, conservation plays a pivotal role in protecting both wildlife and human health through the maintenance of balanced, resilient ecosystems [2]. Italy reflects this general trend, with many native mammal populations declining or increasingly confined to protected areas such as national parks and reserves. Tuscany hosts a high density of wild mammals and harbours the largest population of wild ungulates in peninsular Italy, estimated at approximately 400,000 individuals, with steadily increasing numbers.

The region accounts for about 40% of the national roe deer population, 45% of fallow deer, and 30% of wild boar [3]. Among the multiple stressors affecting wildlife populations, infectious diseases have gained increasing recognition as important drivers of biodiversity decline and as potential threats to both animal and human health [1,4]. Many pathogens circulate silently within wildlife without causing evident disease, yet they can spillover into domestic animals or humans under favourable ecological or environmental conditions [5,6]. Such cross-species transmission events can alter ecosystem dynamics, as demonstrated by viral outbreaks in wild carnivores and ungulates that have led to population declines or secondary infections in domestic livestock. Conservation and infectious diseases are closely interconnected. Understanding the distribution and diversity of pathogens circulating in wildlife is critical for both conservation biology and public health preparedness, in line with the One Health concept that highlights the interdependence of animal, human, and environmental health [7]. In this context monitoring wildlife health remains challenging because free-ranging animals are difficult to capture or handle. Traditional surveillance methods—including live trapping, carcass collection, or sampling during hunting and culling—often provide limited coverage and may bias results toward diseased or dead individuals [8,9]. Moreover, direct capture can induce stress or injury, particularly in sensitive or endangered species, and is logistically demanding in remote areas. These constraints hinder large-scale or long-term monitoring programs, limiting the capacity to detect early signs of emerging infections or ecosystem-level viral circulation.

To overcome these limitations, non-invasive sampling methods, such as the analysis of feces, urine, hair, or environmental swabs, have emerged as valuable alternatives for wildlife surveillance. Fecal samples, in particular, can be collected without disturbing animals and yield valuable information. They allow the identification of pathogens shed through the gastrointestinal tract and—especially in predators and scavengers—can also reveal viruses acquired from prey species, thus providing insight into both host-specific enteric viruses and environmentally acquired pathogens [10,11]. This sampling methodology enables the collection of data from animals that cannot be captured or directly handled and are widely used in studies on both domestic and wild animals. On the other hand, the possible environmental viral contamination even on fresh samples cannot be excluded. Concerning this particular aspect of the sampling procedure adopted, the aim of this research is to define the presence of viruses in the environment rather than in a single host. Numerous investigations have analyzed viruses from feces or rectal swabs collected from wild animals that were either live-captured, found dead, or culled through management programs using either targeted molecular assays or broader metagenomic approaches for virome characterization [12,13,14,15]. Thus, fecal-based studies can reflect not only the health of individual hosts but also the broader virological landscape of the ecosystem.

The introduction of next-generation sequencing (NGS) and metagenomic techniques has revolutionized the study of the wildlife virome, enabling the detection of both known and novel viral genomes without prior assumptions [16]. Metagenomic analysis is a well-established methodology with broad detection capabilities and applicability to various biological matrices, making it a valuable tool for exploring samples potentially containing unexpected or novel viruses [17]. These methods have revealed remarkable viral diversity across taxa and geographical regions: novel members of the *Parvoviridae* and *Hepeviridae* family in red foxes in the Netherlands [12], diverse *Astroviridae* members in wild boars and cervids [18,19,20], and novel *Picornaviridae* and *Picobirnaviridae* in small carnivores and rodents [21,22]. In Europe, however, studies encompassing multiple sympatric species within the same ecosystem remain scarce, leaving significant gaps in our understanding of viral circulation and host–pathogen interactions.

The present research investigates the fecal virome of wild mammals inhabiting the Foreste Casentinesi National Park (Central Italy)—one of the country’s most biodiverse and least anthropized ecosystems. By integrating conventional polymerase chain reaction (PCR) and metagenomic NGS approaches, this study aims to (i) characterize the diversity of viruses circulating among wild mammals, (ii) identify potentially novel or divergent viral lineages, and (iii) evaluate the effectiveness of non-invasive sampling as a model for ecosystem-level viral surveillance. Strengthening wildlife pathogen monitoring through such integrated methods may enhance early detection of emerging threats and support both biodiversity conservation and public health within the One Health framework [4].

## 2. Materials and Methods

### 2.1. Foreste Casentinesi National Park (PNFC)

**Figure 1:**
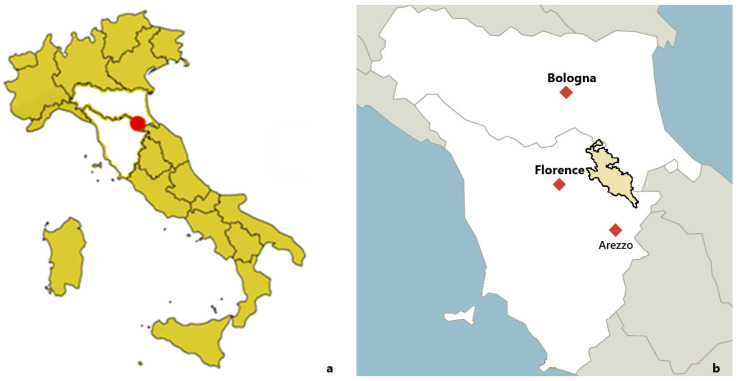
The PNFC sampling area, located in Tuscany and Emilia-Romagna (**a**) is shown in light yellow color (**b**).

The Foreste Casentinesi National Park (PNFC) covers an area of 368 km^2^ across two regions, Emilia-Romagna and Tuscany, with elevations ranging from 400 to 1658 meters above sea level. Forests account for over 80% of the Park’s total surface area. The extensive woodland coverage, the presence of diverse habitats and vegetation types, along with low human population density, make the Park an ideal environment for the presence and spread of wildlife, which is notable for its richness and diversity of species. For the purposes of this study, only the portion of the Park located within the Province of Arezzo (138 km^2^) was considered (Figure 1). The Casentinesi Forests National Park represents a crucial stronghold for the conservation of mammalian biodiversity in the northern Apennines. Its extensive mature woodlands and low levels of human disturbance support a wide range of wild species, including several conservation concerns. Among large mammals, the Italian wolf (*Canis lupus italicus*) has successfully recolonized the area, yet remains vulnerable due to habitat fragmentation, hybridization, and human–wildlife conflict. The park also hosts the European wildcat (*Felis silvestris silvestris*), a rare and threatened species in Italy, endangered by hybridization with domestic cats and by the reduction in continuous forest cover. Overall, the Casentinesi Forests National Park serves as a key refuge for endangered mammalian species and a model area for long-term biodiversity management within the Apennine ecosystem (https://www.parcoforestecasentinesi.it/) (accessed on 3 September 2025).

### 2.2. Sampling

Sampling activities were carried out following an agreement between the Department of Veterinary Sciences of the University of Pisa and the Foreste Casentinesi, Monte Falterona and Campigna National Park Authority (26 February 2021). The study area, corresponding to the portion of the Park located within the Province of Arezzo, was divided into eleven cells measuring five × five Km. Each transect of eight-1ten Km length was surveyed at least two times simultaneously by two–three operators, who followed and examined both the main trail/path and the surrounding area, considering a buffer zone of approximately ten m on each side. Additionally, any clearings, openings along or adjacent to the main route, as well as clearly identifiable animal paths, were carefully inspected. Given the aims of the study, only fresh fecal samples from wild mammals were collected. The sampling sessions were conducted between March 2021 and June 2022. Fecal samples were attributed to species based on morphological characteristics (appearance, size), deposition pattern, and content. Freshness was assessed based on consistency, color, moisture, mucus, and odor [23]. All samples were collected using sterile gloves (EN455) (CliniSafe Ltd., Lancaster, UK), aliquoted into Eppendorf tubes, and stored at −80 °C within six hours of collection.

### 2.3. Sample Processing

Samples were primarily analyzed in pools for conventional PCR screening and subsequent NGS analysis. Pools consisting of three to four fecal samples were created by grouping individual samples belonging to the same species from the same or neighboring transects. In details 100 mg per sample were ground and homogenized together until a uniform mixture was obtained, from which 200 mg were taken for extraction. Nucleic acid extraction was performed using the AllPrep PowerFecal DNA/RNA Kit (Qiagen, Hilden, Germany) according to the manufacturer’s instructions, including optional on-column DNase and RNase digestion. After lysis and clarification, an additional filtration step was performed using 0.45 μm filters (Euroclone, Milan, Italy). Extracted nucleic acids were quantified using a Nanodrop spectrophotometer (Thermofisher scientific, Waltam, MA, USA), aliquoted, and stored at −80 °C until further analysis.

#### PCR Screening

Conventional PRC screening was performed on sample pool targeting the major fecal-shed viruses. Based on an extensive literature review, species-specific viral target panels were developed, while for less-studied species such as the porcupine (*Hystrix cristata*), badger (*Meles meles*), and small mustelids, broader panels were designed to include viruses from phylogenetically related taxa as well (Appendix A).

When possible—particularly for species with limited or no virological data—broad-range PCR protocols were also employed [2,24,25,26,27]. This strategy maximized the information obtained from each sample and provided an indirect means of investigating elusive species that are difficult or impossible to sample directly. Primer sequences used in this study, along reference details are provided in Table 1.

For the detection of viral DNA targets, conventional PCR was performed following literature protocols as presented in Table 1 using the Wonder Taq Hot Start Kit (Euroclone, Milan, Italy), while RNA viruses were analyzed via RT-PCR using the OneStep RT-PCR Kit (Qiagen, Hilden, Germany). For the second round of nested or semi-nested protocols, Wonder Taq Hot Start was again employed. All reactions were set up following the manufacturers’ protocols.

PCR products were subjected to agarose gel electrophoresis to verify the presence of the amplicons of expected size. Samples showing bands of the correct size were cut from gel and purified using the EuroSAP PCR Enzymatic Clean-up Kit (Euroclone, Milan, Italy) prior to sequencing.

Sanger sequencing was performed by BMR Genomics (Padua, Italy). Resulting sequences were analyzed using the online BLAST (2.17.0 version) tool to confirm target identity choosing somewhat similar sequence (blastn) algorithm using core nucleotide database (core nt). High-quality sequences were further processed using BioEdit (5.0.9 version) and submitted to GenBank.

### 2.4. Metagenomics Analysis

Although PCR using consensus primers is a useful tool for characterizing viral populations, enabling the detection of viruses even distantly related to known ones, this approach remains limited by the need for primer design and the selection of targets by the operator. To overcome these constraints and reduce operator bias, we employed a next-generation sequencing (NGS) approach, which does not rely on prior knowledge of viral sequences.

Four pooled fecal samples from red foxes (*Vulpes vulpes*) were subjected to metagenomic analysis to characterize the fecal virome. To enable unbiased amplification of viral nucleic acids, both RNA and DNA extracted from each pool underwent Sequence-Independent Single-Primer Amplification (SISPA) [43,44].

Following SISPA and quantification using a Qubit fluorometer and the dsDNA Assay Kit (Thermo Fisher Scientific, Waltam, MA, USA), RNA- and DNA-derived products were combined in a 1:1 ratio and submitted to IGA Technology Services Srl (Udine, Italy) for shotgun metagenomic sequencing. Libraries were prepared and sequenced on the Illumina NovaSeq 6000 platform (Illumina, Madison, WI, USA), generating approximately 30 million 150 bp paired-end reads per sample.

### 2.5. Bioinformatics

A preliminary taxonomic classification of the sequencing reads was performed by IGA Technology Services using Kraken v.2, a high-speed and accurate program for taxonomic assignment of DNA sequences [45].

Subsequently, a more detailed bioinformatic analysis was conducted using Geneious Prime^®^ 2022.2.2), as indicated in Pacini et al. [46] supported by computing resources provided by the University of Pisa Data Center. Analyses were carried out on a 64-bit Windows-based virtual machine equipped with dual Intel Xeon Gold 5120 CPUs (2.20 GHz) and 128 GB RAM.

### 2.6. Phylogenetic Analysis

Phylogenetic relationships were inferred using the Maximum Likelihood (ML) method under the Tamura–Nei substitution model. The initial tree for the heuristic search was selected by choosing the tree with the superior log-likelihood between a Neighbor-Joining (NJ) tree [47] and a Maximum Parsimony (MP) tree [48]. The NJ tree was generated from a matrix of pairwise distances computed using the p-distance method. The MP tree with the shortest length was selected among ten independent MP searches, each initiated with a randomly generated starting tree. All evolutionary analyses were conducted in MEGA version 12 [49].

## 3. Results

### 3.1. Sample Collection and Pool Preparation

During sampling session, a total of 99 fecal samples were collected along 21 transects. Fifteen samples were excluded during processing due to advanced degradation or uncertain species attribution.

Samples were grouped into 26 pools: two pools of roe deer, four of fallow deer, three of red deer, four of red foxes, four of wolves, four of badgers, four of small mustelids, and one of porcupines.

### 3.2. PCR Results

PCR performed on the nucleic acids extracted from fecal samples pool returned with the following results: out of the 26 fecal pools tested, ten pools (38.5%) were positive for at least one viral target. Viral RNA or DNA were detected in multiple species, including red deer (two/three pools), red foxes (three/four pools), wolves (two/four pools), small mustelids (two/four pools), and porcupine (one/one pool). In contrast, all pools from roe deer, fallow deer, and badgers tested negative for the entire panel of viral targets.

Among the viruses investigated, *Astrovirus* spp. was the most frequently detected, found in red deer (two/three pools), red foxes (three/four pools), wolves (one/four pools), small mustelids (one/four pools), and porcupine (one/one pool). Several additional viruses were identified in red foxes, including bocavirus, *Canine parvovirus* (CPV), *Kobuvirus, Adenovirus type* 1 (CAdV-1), and *Coronavirus*, each detected in one pool. In wolves, *Kobuvirus* was also found in one pool, while adenovirus was the only additional virus detected in small mustelids (two/four pools).

No pools tested positive for *Canine distemper virus* (CDV), Bovine viral diarrhea virus (BVDV), *Bovine papillomavirus* (BPV), *Canine circovirus*, or *Torque teno viruses* (TTV1 and TTV2).

A detailed analysis of the positive pools revealed specific associations between viral targets and host species. Astrovirus was the most frequently detected virus, observed in multiple species. Several other viral agents were identified in red foxes, wolves, and small mustelids.

#### 3.2.1. GenBank Submission and BLAST Analysis

All confirmed positive PCR amplified sequences were submitted to GenBank. Table 2 lists accession numbers alongside BLAST results, including the closest match, nucleotide identity percentage, and E-value.

#### 3.2.2. Phylogenetic Analysis

**Figure 2:**
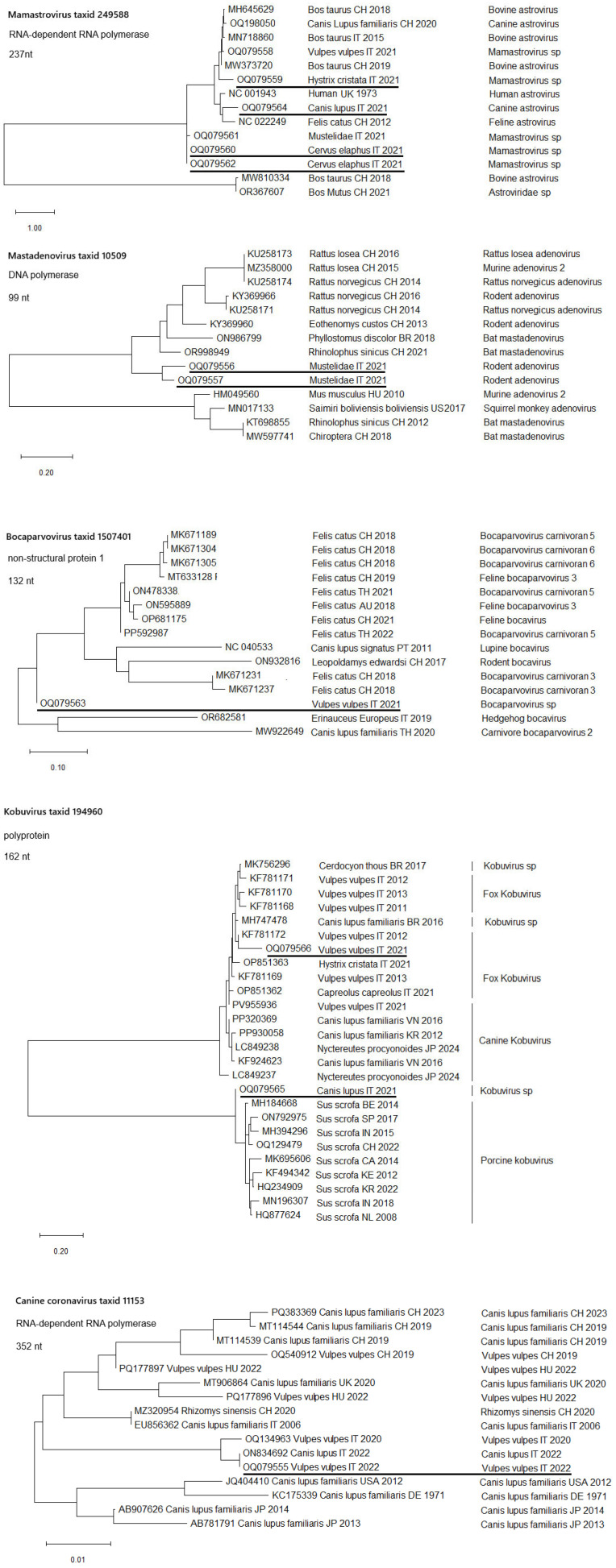
Evolutionary analysis by the Maximum Likelihood method.

Phylogenetic analyses were performed for viral species for which PCR amplification yielded sequences of sufficient length and informative content. The resulting trees (Figure 2) showed that the detected strains clustered within the expected lineages, confirming their taxonomic assignment. For other viruses, the available amplicons were too short or lacked adequate phylogenetically informative regions, preventing reliable analysis.

Phylogenetic analysis of *Mamastrovirus*, *Mastadenovirus*, *Bocaporvovirus*, *Kobuvirus* and *Canine coronavirus* are presented along with their number of nucleotide analysed and protein fragment name. Accession numbers are reported together with the corresponding host species, country of origin, and year of sequence submission. Taxonomic classification follows the information reported in the respective GenBank records. Sequences newly identified in this study are underlined. BLAST analyses were performed for these sequences and were restricted to taxa as indicated in the panel.

### 3.3. Metagenomic Analysis

Metagenomic analysis was performed on 4 red fox pools. Pools were selected based on PCR results, with red foxes chosen due to their high positivity rate and limited virological data in the literature. Table 3 present the total reads, the percentage of classified vs. unclassified reads, and the taxonomic breakdown. Among classified reads, the vast majority (76–98%) belonged to Bacteria (taxid: 2), while viral reads (Viruses, taxid: 10,239) represented 0.4–2%.

#### In-Depth Bioinformatic Analysis

Further analyses were conducted using Geneious Prime^®^, aiming to refine viral identification from the metagenomic datasets. Sequencing reads were compared against two custom viral databases constructed through the NCBI Virus portal: one comprising all known viral sequences isolated from mammals (TaxID: 40674, excluding *Homo sapiens* viral sequence not related to zoonotic potential, as of 21 October 2022), and another including viruses known to infect birds (TaxID: 8782). The bird database was included to identify potential avian viruses circulating within wild bird populations, considering that the sampled material could have originated from bird predators. The mammalian virus database contained 470,053 sequences and 1033 complete reference genomes, while the avian virus database included 297,656 sequences and 212 complete reference genomes.

Reads were aligned to the reference genomes within these databases to generate consensus sequences, each representing a potential viral fragment identified in the sample. For each consensus sequence, the following parameters were recorded: total consensus length, percentage of reference genome coverage, number of identical sites, pairwise identity, and BLAST E-value.

To ensure reliability and reduce background noise, only consensus sequences meeting strict filtering criteria were retained for further interpretation. Specifically, retained sequences were required to be longer than 150 base pairs, to present more than 75% identical sites, a pairwise identity above 80%, and a BLAST E-value less than or equal to 10^−100^. [46]. These thresholds were set to minimize false positives and to focus the analysis on highly confident viral identifications. Sequences that did not match the reference sequences present in the database were discarded. Non-matching sequences were excluded based on the comparison with the database.

Table 4 presents these results in detail.

## 4. Discussion

For the aim of this research a systematic sampling design was implemented across the PNFC, based on predefined transects. This systematic framework, combined with a pooling strategy, optimized data collection and enabled an ecosystem-level assessment of viral circulation throughout the park, ensuring even spatial coverage of the study area. The park’s high biodiversity, ecological continuity, heterogeneous habitats, and limited anthropogenic disturbance made it particularly suitable for this approach. The main limitations concerned the quality and freshness of fecal samples, which were influenced by environmental factors such as temperature, precipitation, snow cover, and leaf litter. Deposition sites also affected sample integrity, particularly in relation to potential environmental contamination. However, such contamination was not considered a limiting factor in this context, as the study aimed to characterize the ecosystem virome rather than evaluate the health status of individual animals. In our study, viral pathogens strictly associated with *Homo sapiens* were excluded, as they are not relevant to the natural virome dynamics within the study area. However, zoonotic agents potentially shared between wildlife and humans were retained, given their ecological and epidemiological importance at the wildlife–human interface. The inclusion of the bird database was motivated by the need to obtain a comprehensive overview of the ecosystem virome. Considering that many mammalian predators regularly prey upon avian species, this approach allowed the detection of viral sequences potentially originating from *Aves*, thereby providing a more complete picture of viral diversity across trophic levels.

The integration of conventional PCR screening with metagenomic next-generation sequencing (NGS) enhanced viral detection, combining the high sensitivity of targeted assays with the broad exploratory capacity of untargeted methods. This dual approach provided a more comprehensive understanding of viral diversity in wildlife. Red foxes, being widespread and opportunistic feeders proved particularly informative, confirming their role as sentinel species for ecosystem and zoonotic health surveillance [5,7,12,50,51].

The viruses identified belonged mainly to the families *Astroviridae*, *Picornaviridae* (*Kobuvirus*), *Parvoviridae* (CPV, *Bocavirus*, AAV), *Adenoviridae*, *Circoviridae*, *Anelloviridae*, and *Picobirnaviridae*. The main findings for each viral family are discussed below.

### 4.1. Astroviridae

Astroviruses (family *Astroviridae*) are viruses detected in a wide range of domestic and wild hosts. They are classically associated with gastroenteritis, especially in young animals, but recent studies have also reported extra-intestinal infections, such as encephalitis and respiratory involvement, and a potential for cross-species transmission [46,52]. In the Tuscany region, which is characterized by high wildlife biodiversity and a dense population of wild ungulates, metagenomic investigations have identified astrovirus as one of the most frequently detected viral agents in fecal samples from wild mammals, including wild boar, foxes, and mustelids [46]. These findings suggest that Tuscan wildlife may act as an important reservoir of astroviruses, with implications for wildlife health surveillance, zoonotic risk assessment, and conservation. Although the full pathogenic and epidemiological roles of astroviruses in natural environments remain unclear, their detection across multiple species supports their relevance within a One Health framework. Astrovirus was the most widespread virus detected, found in deer, foxes, wolves, porcupines, and small mustelids. These findings expand the known host range in Europe [19,20,53,54,55]. Although some sequences may reflect dietary origin, sequences obtained from fox pools showed high homology with *Bovine astrovirus*, the consistent detection across taxa supports the presence of astroviruses in the environment and highlights their ecological importance in wildlife viral dynamics.

To date, there are no published reports of *Astrovirus* infection in wolves, European porcupines (*Hystrix cristata*), or the small mustelid species sampled, including weasel (*Mustela nivalis*), beech marten (*Martes foina*), pine marten (*Martes martes*), and polecat (*Mustela putorius*), making our findings the first documented detections in these species.

In general, the relatively low sequence identity with known astrovirus strains suggests the circulation of divergent or novel lineages within the PNFC ecosystem.

### 4.2. Picornaviridae

Kobuviruses (genus *Kobuvirus*, family *Picornaviridae*) are non-enveloped, single-stranded positive-sense RNA viruses that have been detected in a wide range of domestic and wild mammals and are increasingly recognized as enteric pathogens. These viruses have been associated with diarrhoea in cattle, pigs, goats and wildlife, and molecular surveys in Italy have detected kobuvirus RNA in wild ungulates including roe deer, suggesting a multi-host interface and possible wildlife reservoir role [56]. Although the clinical impact in many wildlife species remains poorly defined, their detection in overlapping habitats of livestock and wild fauna underscores their relevance to animal health, surveillance and conservation.

Kobuviruses were detected in foxes and wolves, consistent with previous Italian reports [56,57,58,59,60].

In our study, sequences obtained in foxes by positive PCR analysis showed high nucleotide identity with *Canine kobuvirus* (CaKoV), particularly with those previously identified in Italian foxes.

The wolf kobuvirus sequence showed high similarity to porcine kobuvirus, suggesting dietary origin and implying the circulation of this virus in wild suids. The detection of kobuviruses in multiple carnivore species supports their broad host range and highlights predator-prey viral transmission as an important pathway for viral maintenance in natural ecosystems.

### 4.3. Adenoviridae

Adenoviruses (family *Adenoviridae*) are viruses that infect a broad spectrum of vertebrate hosts, including mammals, birds, and reptiles. They are typically associated with respiratory, ocular, and gastrointestinal infections, though some species can cause systemic disease and mortality in wildlife populations. Molecular studies in Europe have identified adenovirus DNA in several wild mammals, including deer, wild boar, and carnivores, suggesting a wide host range and possible interspecies transmission [2]. In Italy, adenoviruses have been detected in wild ungulates and carnivores, including samples from Tuscany, where viromic analyses highlighted their presence in fecal material from hospitalized wildlife [46].

By metagenomic analyses and PCR assays using *Adenovirus* spp generic primers, sequences related to *Squirrel adenovirus* (SqAdV) and rodent *Mastadenovirus* (MAdV), were detected.

In recent years, SqAdV has gained increasing relevance due to the expansion of its geographical range across several European regions and its impact on the health of the Eurasian red squirrel (*Sciurus vulgaris*), with significant implications for conservation and reintroduction programs targeting this protected species [61,62,63,64,65,66]. The presence of squirrel adenovirus (SqAdV-1) in fox feces likely reflects predation but has conservation relevance for the local red squirrel population, representing a potential warning sign for the red squirrel population inhabiting the study area. Such detection may highlight a health issue that is difficult to identify by other means, as the infection does not typically produce easily recognizable clinical signs and, in natural environments, carcasses of deceased animals are rapidly removed by predators or scavengers. The detection of rodent-like adenoviruses in mustelids [67] may also indicate dietary origin or potential host adaptation, underlining the value of metagenomics for detecting cross-species viral events.

### 4.4. Parvoviridae

Parvoviruses (family *Parvoviridae*, subfamily *Parvovirinae*) are small, non-enveloped, single-stranded DNA viruses infecting a wide range of vertebrate hosts. They are known for their high environmental stability and for causing severe diseases, including enteritis, myocarditis, and reproductive disorders, particularly in carnivores and ungulates [31,46]. Recent molecular investigations have revealed the circulation of diverse parvovirus lineages in European wildlife, including red foxes (*Vulpes vulpes*), wild boar (*Sus scrofa*)*,* and roe deer (*Capreolus capreolus*), indicating the presence of sylvatic reservoirs. In Italy, and specifically in Tuscany, parvoviruses have been detected through metagenomic analyses of fecal samples from hospitalized wild mammals [46].

Foxes carried multiple parvoviruses—*Canine parvovirus* (CPV-2c), Bocavirus, and Adeno-Associated virus (AAV)—highlighting frequent viral exchanges between wild and domestic carnivores [12,31,68,69,70,71,72,73,74]. The co-detection of adenoviruses and AAV within the same pool suggests possible co-infection, while the divergent bocavirus sequences indicate continued viral diversification among wild carnivores. These findings emphasize the interface between domestic and wildlife populations as key zones for viral evolution and spillover. Within the *Parvoviridae* family, a sequence corresponding to Fox adeno-associated virus, a novel adeno-associated virus (AAV) was identified in a fox fecal sample by NGS methodology [12]. Fox AAV was detected in a pool in which two adenoviruses (*Squirrel adenovirus* and an *Aviadenovirus*) were also present; it would therefore be of interest to determine whether these viruses occurred in the same individual and whether Fox AAV was actively replicating. Several AAV serotypes have been isolated from birds and a wide range of mammalian species, including humans, but they have not been associated with clinical disease [75]. *Bocaparvovirus* is a member of the *Parvovirinae* subfamily and has been reported in humans as well as in a wide range of domestic and wild species [76,77,78,79,80]. In our study, the sequence obtained displayed significant homology with both Feline bocaparvovirus 3 and Lupine bocavirus, suggesting the circulation of divergent strains in wild carnivores and warranting further investigations for accurate classification.

Sequences belonging to the families *Circoviridae*, *Anelloviridae*, and *Picobirnaviridae* were also identified, mostly in fox feces. Many of these sequences likely originated from dietary or environmental sources rather than active infections. Nevertheless, their presence underscores the complex viral landscape within the PNFC. Viruses such as torque teno viruses and porcine circovirus-like sequence may serve as environmental indicators or markers of interspecies viral exchange within the ecosystem.

Our results reveal notable differences compared with recent studies on wildlife admitted to the Veterinary Teaching Hospital of the University of Pisa after recovery from the surrounding province [46,81]. In the Pisa area, fewer viral taxa were detected, but with higher prevalence rates, whereas in the PNFC we found a wider viral diversity, though at lower prevalence. These contrasting patterns can be explained by the ecological characteristics of the two study areas. The province of Pisa has lower biodiversity and highly fragmented habitats, where wild animals live at higher population densities. This situation may promote the circulation and higher prevalence of specific pathogens. In contrast, the PNFC hosts greater biodiversity and large, continuous habitats, which likely allow the coexistence of multiple viral species but limit their widespread transmission.

A notable difference emerged between conventional PCR and metagenomic sequencing applied to the same samples. Several viral targets detected by PCR were not confirmed by metagenomics, reflecting the methodological differences between the two approaches. PCR remains highly sensitive and specific for known targets, whereas metagenomic workflows, such as SISPA, rely on random amplification that can bias genome coverage and favor abundant viral genomes, thus limiting the recovery of complete or less represented sequences [82].

Despite these limitations, metagenomics proved to be a powerful complementary approach, allowing the detection of unexpected or previously unreported viruses that would have been missed by targeted PCR screening. The combined use of conventional and next-generation molecular methods expanded the range of detectable viruses, enabling the identification of both known pathogens—sometimes underrepresented in the population—and novel or unexpected viral agents. Interestingly, most viral sequences were obtained from carnivorous or scavenging species. This pattern likely reflects not only their own viral infections but also those of their prey or other sympatric species. The greater number of viral sequences found in carnivores and scavengers likely reflects their feeding ecology. As predators and consumers of carcasses, these species are in frequent contact with tissues and bodily fluids of various prey species, which may harbor diverse viruses. Consequently, viral sequences derived from prey can be detected in their fecal samples, contributing to the apparent higher viral diversity observed in these trophic groups. These findings highlight the ecological value of the selected sample type, which provided insights into pathogens affecting the sampled hosts as well as those circulating more broadly within the ecosystem. The detection of viruses typically associated with domestic animals in wildlife emphasizes the permeability of ecological boundaries and underscores the importance of continued surveillance within a One Health framework. Non-invasive monitoring of wildlife viromes provides early warning signals for emerging zoonoses and contributes to biodiversity conservation by revealing ecosystem changes that influence host–pathogen dynamics. Strengthening such approaches in protected areas can help anticipate and mitigate the risk of disease emergence.

## 5. Conclusions

This study demonstrated the effectiveness of a non-invasive approach based on environmental fecal sampling for virological surveillance of wildlife in a high-biodiversity ecosystem such as the PNFC. The integration of conventional molecular techniques and metagenomic analyses enabled the detection of a wide range of viruses, including known pathogens, emerging viruses, and agents not previously reported in certain host species or in Italy. The findings confirm the central role of carnivorous and scavenging species, such as the red fox, as ecological sentinels capable of reflecting viral circulation at the community level. The presence of viruses typically associated with domestic species suggests potential interactions between wildlife and anthropogenic environments, with implications for both animal and public health. Moreover, the detection of viruses considered non-pathogenic or of unknown impact highlights the need to deepen our understanding of host–virus dynamics in natural settings. From a One Health perspective, ongoing monitoring of the wildlife virome represents a crucial tool for anticipating and mitigating the risks associated with the emergence of new infectious diseases.

## Figures and Tables

**Table 1:** 5′-3′ primer sequences used for PCR analysis. Abbreviations for each virus are listed at the end of the manuscript.

	5′-3′ Primer Forward	5′-3′ Primer Reverse	Reference
*Adenovirus* spp.	1° round: TNMGNGGNGGNMGNTGYTAYCC 2° round: GTDGCRAANSHNCCRTABARNGMRT	1° round: GTDGCRAANSHNCCRTABARNGMRTT 2° round: CCANCCBCDRTTRTGNARNGTRA	[28]
*CAdV 1,2*	CGCGCTGAACATTACTACCTTGTC	CCTAGAGCACTTCGTGTCCGCTT	[29]
*CPV*	ACAAGATAAAAGACGTGGTGTAACTCAA	CAACCTCAGCTGGTCTCATAATAGT	[30]
*FPV/CPV*	ACAAGATAAAAGACGTGGTGTAACTCAA	CAACCTCAGCTGGTCTCATAATAGT	[31]
*Bocavirus* spp.	GCCAGCACNGGNAARACMAA	CATNAGNCAYTCYTCCCACCA	[32]
*CBov 1*	1° round: CARTGGTAYGCTCCMATYTTTAA 2° round: TGGTAYGCTCCMATYTTTAAYGG	1° round: TGGCTCCCGTCACAAAAKATRTG 2° round: GCTCCCGTCACAAAAKATRTGAAC	[33]
*CBoV 2*	AGGTCGGCCACTGGCTGT	CAGCTTAACGGCATTCACTA	[32]
*CBoV 3*	1° round: CAGATTTGGGGGTCCTGCAT 2° round: ATGCCGTCACCAATCCACAT	1° round: GCACTGTCTGCGCTGAAAAA 2° round: AGCTTGTGGTGGACAGTAGC	[34]
*LBoV*	AGACCAGATGCTCCACATGG	TGCCTGCCACGGATTGTACC	[35]
*Canine CV*	CTGAAAGATAAAGGCCTCTCGCT	AGGGGGGTGAACAGGTAAACG	[36]
*TTV1*	CGGGTTCAGGAGGCTCAAT	GCCATTCGGAACTGCACTTACT	[37]
*TTV2*	TCATGACAGGGTTCACCGGA	CGTCTGCGCACTTACTTATATACTCTA	[37]
*Kobuvirus* spp.	TGGAYTACAAGTGTTTTGATGC	ATGTTGTTRATGATGGTGTTGA	[38]
*Astrovirus* spp.	1° round a: GARTTYGATTGGRCKCGKTAYGA 1° round b: GARTTYGATTGGRCKAGGTAYGA 2° round a: CGKTAYGATGGKACKATHCC 2° round b: AGGTAYGATGGKACKATHCC	GGYTTKACCCACATNCCRAA	[39]
*Coronavirus* spp.	1° round: GGKTGGGAYTAYCCKAARTG 2° round: GGTTGGGACTATCCTAAGTGTGA	1° round: TGYTGTSWRCARAAYTCRTG 2° round: CCATCATCAGATAGAATCATCAT	[40]
*BVDV*	ATGCCCWTAGTAGGACTAGCA	TCAACTCCATGTGCCATGTAC	[41]
*Bopivirus* spp.	CTGRGCAAGTTCACCAACAA	GTCCATGACAGGGTGAATCA	[42]
*CDV*	ACTTCCGCGATCTCCACTGG	GCTCCACTGCATCTGTATGG	[35]

**Table 2:** Genbank accession numbers alongside BLAST results, including the closest match, nucleotide identity percentage, and E-value.

Virus	Host	Genbank	Reference Sequence	Ident % (QueryCov%)	E Value
CPV	Red Fox	CPV/fox/169/IT OQ079554	*Canine parvovirus 2c* MF177262.1	84,83% (97%)	6e-31
CCoV	Red Fox	CCoV/fox/208/IT OQ079555	*Canine coronavirus* ON834692.1	100.00% (100%)	0.0
AdV	Small mustelids	RAdV/mustelidae/878/IT OQ079556	*Rodent adenovirus* KY369960.1	75.91% (100%)	1e-49
	Small mustelids	RAdV/mustelidae/888/IT OQ079557	*Rodent adenovirus* KY369960.1	74.74% (99%)	1e-42
AstV	Red Fox	Mastv/fox/46/IT OQ079558	*Bovine astrovirus* MW373720.1	90.82% (100%)	7e-155
	Porcupine	Mastv/porcupine/484/IT OQ079559	*Mamastrovirus 3* MH399894.1	91,27% (100%)	2e-156
	Cervo	Mastv/reddeer/707/IT OQ079560	*Murine astrovirus* JQ408746.1	88.49% (99%)	9e-128
	Small mustelids	Mastv/mustelidae/883/IT OQ079561	*Murine astrovirus* JQ408746.1	87.95% (100%)	3e-81
	Red Deer	Mastv/redeer/885/IT OQ079562	*Murine astrovirus* JQ408746.1	88.56% (100%)	7e-129
	Wolf	CAsV/wolf/481/IT OQ079564	*Canine astrovirus* OQ198039	88.80% (100%)	3e-133
BoV	Red Fox	BoV/fox/171/IT OQ079563	*Bocaparvovirus carnivoran5* PP592987	88.55% (99%)	3-e83
KoV	Wolf	Kov/wolf/525/IT OQ079565	*Porcine kobuvirus* MH184668.1	93.30% (99%)	8e-88
	Red Fox	Kov/fox/88/IT OQ079566	*Fox kobuvirus* KF781172.1	92.49% (100%)	5e-63

**Table 3:** Results of the taxonomic classification using Kraken 2.

Pool Number	N° of Sequences	Classified Sequences * (%)	Unclassified Sequences * (%)	Bacteria Sequences * (%)	Viral Sequences * (%)
1	1.86 × 10^7^	21	79	76	2
2	1.79 × 10^7^	29	72	92	1
3	2.17 × 10^7^	26	74	89	0.7
4	2.18 × 10^7^	59	41	98	0.4

The percentage of sequences assigned to Bacteria and Viruses is calculated over the number of classified sequences (*).

**Table 4:** Post data analysis of NGS results.

Pool Number	Genbank	Ref-Seq	n° Reads	n° Nucleotide	Pairwise Identity	E Value
1	PX314117	*Porcine picobirnavirus* MW978536.1	213	467	100%	0.0
	PX314118	*Rodent circovirus* KY370041.1	7	480	89.97%	1e-145
	PX314119	*Anelloviridae* spp. MF346362.1	2	427	99.77%	0.0
2	PX314120	*Fox adeno-associated virus* KC878874	28	1288	100%	0.0
	PX314121 PX314122 PX314123	*Red squirrel adenovirus-1* NC_035207	27	2129	69.66% 88.58% 100	1e-24 1e-177 0
	PX314124	*Pigeon adenovirus* NC024474	17	319	100%	2e-104
	PX314125 PX314126 PX314127	*Torqueteno felis virus* MT538010	25	779	92.21% 98.06% 70.71%	4e-51 7e-67 2e-30
3	PX314128 PX314129	*Porcine circo-like virus* JF713717	31	1759	90.90% 98.15%	0.0 0.0

## Data Availability

The original contributions presented in this study are included in the article. Further inquiries can be directed to the corresponding author.

## Abbreviations

CPV	Canine parvovirus
CAdV-1	Canine adenovirus type 1
CDV	Canine distemper virus
BVDV	Bovine viral diarrhea virus
BPV	Bovine papillomavirus
CCoV	Canine circovirus
TTV1 and TTV2	Torque teno viruses
AdV	Adenovirus
RAdV	Rodent Adenovirus
MAdV	Mastadenovirus
PMWS	Postweaning multisystemic wasting syndrome
RMAdV	Rodent Mastadenovirus
SqAdv	Squirrel adenovirus
CAsV	Canine astrovirus
BoV	Bokaviru
KoV	Kobuvirus
CaKoV	Canine Kobuvirus
CcAstV	Capreolus capreolus astrovirus
WMS	Wet Mink Syndrome
LAdV-1	Lutrine adenovirus
PBV	Picobirnavirus
PCV	Porcine circovirus
PNFC	Parco Nazionale Foreste Casentinesi

## Appendix A

Bibliographic reference of studied viruses

	**AdV**	**PV**	**BoV**	**CV**	**TTV**	**V**	**AstV**	**CoV**	**PeV**	**BPV**	**MoV**	**Ref.**
Roe deer	*AdV* spp.	-	*BPV* spp.	-	-	*KoV* spp.	*AstV* spp.	*CoV* spp.	*BVDV*	*BPV* spp.	-	[19,53,83,84,85,86,87,88,89,90,91]
Fallow deer	*AdV* spp.	-	*BPV* spp.	-	-	*KoV* spp.	*AstV* spp.	*CoV* spp.	*BVDV*	*BPV* spp.	-	
Red deer	*AdV* spp.	-	*BPV* spp.	-	-	*KoV* spp.	*AstV* spp.	*CoV* spp.	*BVDV*	*BPV* spp.	-	
Red fox	*CAdV 1,2* *AdV* spp.	*CPV* *FPV*	*CBoV 1,2,3* *BPV* spp.	*CCV*	-	*KoV* spp.	*AstV* spp.	*CoV* spp.	-	-	*CDV*	[12,24,54,57,69,92,93,94,95,96,97,98,99]
Wolf	*CAdV 1,2* *AdV* spp.	*CPV* *FPV*	*LBoV* *CBoV 1,2,3* *BPV* spp.	*CCV*	-	*KoV* spp.	*AstV* spp.	*CoV* spp.	-	-	*CDV*	[24,58,68,69,93,99,100,101]
Badger	*CAdV 1,2* *AdV* spp.	*CPV* *FPV*	*BPV* spp.	-	*TTV 1,2*	*KoV* spp.	*AstV* spp.	*CoV* spp.	-	-	*CDV*	[67,69,76,92,95,102,103,104,105,106,107,108,109]
Small mustelids	*CAdV 1,2* *AdV* spp.	*CPV* *FPV*	*BPV* spp.	-	*TTV 1,2*	*KoV* spp.	*AstV* spp.	*CoV* spp.	-	-	*CDV*	
Porcupine	*AdV* spp.	-	*BPV* spp.	-	-	*KoV* spp.	*AstV* spp.	*CoV* spp.	-	-	-	[14,54,66,104,110,111]
